# Genomic profiling of newly diagnosed glioblastoma patients and its potential for clinical utility – a prospective, translational study

**DOI:** 10.1002/1878-0261.12790

**Published:** 2020-09-18

**Authors:** Dorte S. Nørøxe, Christina W. Yde, Olga Østrup, Signe R. Michaelsen, Ane Y. Schmidt, Savvas Kinalis, Mathias H. Torp, Jane Skjøth‐Rasmussen, Jannick Brennum, Petra Hamerlik, Hans S. Poulsen, Finn C. Nielsen, Ulrik Lassen

**Affiliations:** ^1^ Department of Radiation Biology Rigshospitalet Copenhagen Denmark; ^2^ Department of Oncology Rigshospitalet Copenhagen Denmark; ^3^ Center for Genomic Medicine Rigshospitalet Copenhagen Denmark; ^4^ Biotech, Research and Innovation Centre (BRIC) University of Copenhagen Copenhagen Denmark; ^5^ Department of Neuro Surgery Rigshospitalet Copenhagen Denmark; ^6^ Danish Cancer Society Copenhagen Denmark

**Keywords:** chromosomal instability, genomic profiling, newly diagnosed glioblastoma, NTRK fusion, translational relevance, tumor mutational burden

## Abstract

Glioblastoma (GBM) is an incurable brain tumor for which new treatment strategies are urgently needed. Next‐generation sequencing of GBM has most often been performed retrospectively and on archival tissue from both diagnostic and relapse surgeries with limited knowledge of clinical information, including treatment given. We sought to investigate the genomic composition prospectively in treatment‐naïve patients, searched for possible targetable aberrations, and investigated for prognostic and/or predictive factors. A total of 108 newly diagnosed GBM patients were included. Clinical information, progression‐free survival, and overall survival (OS) were noted. Tissues were analyzed by whole‐exome sequencing, single nucleotide polymorphism (SNP) and transcriptome arrays, and RNA sequencing; assessed for mutations, fusions, tumor mutational burden (TMB), and chromosomal instability (CI); and classified into GBM subgroups. Each genomic report was discussed at a multidisciplinary tumor board meeting to evaluate for matching trials. From 111 consecutive patients, 97.3% accepted inclusion in this study. Eighty‐six (77%) were treated with radiation therapy/temozolomide (TMZ) and adjuvant TMZ. One *NTRK2* and three *FGFR3‐TACC3* fusions were identified. Copy number alterations in *GRB2* and *SMYD4* were significantly correlated with worse median OS together with known clinical variables like age, performance status, steroid dose, and O6‐methyl‐guanine‐DNA‐methyl‐transferase status. Patients with CI‐median or TMB‐high had significantly worse median OS compared to CI‐low/high or TMB‐low/median. In conclusion, performing genomic profiling at diagnosis enables evaluation of genomic‐driven therapy at the first progression. Furthermore, TMB‐high or CI‐median patients had worse median OS, which can support the possibility of offering experimental treatment already at the first line for this group.

## Graphical abstract

Landscape of somatic copy number alterations, mutations, and fusions in selected genes in newly diagnosed glioblastoma patients. The most frequent aberrations occurred in *PTEN, CDKN2A/B, EGFR, RB1,* and *NPAS3,* and the most frequent mutations were in *PTEN, TP53, NF1, RB1,* and *EGFR*. One *NTRK2* fusion and three *FGFR3‐TACC3* fusions were identified.

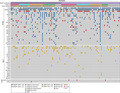

**Abbreviations**
CI: chromosomal instability
EGFR: epithelial growth factor receptor
FFPE: formalin‐fixed paraffin‐embedded
GBM: glioblastoma
GRB2: growth factor receptor bound
IDH: isocitrate dehydrogenase
IT: immunotherapy
LOH: loss of heterozygosity
Mb: megabase
MGMT: O6‐methyl‐guanine‐DNA‐methyl‐transferase
NTRK: neurotrophin tyrosine receptor kinase
OS: overall survival
PFS: progression‐free survival
PS: performance status
RT: radiotherapy
SCA: segmental chromosomal aberration
SCNA: somatic copy number alteration
SMYD4: SET and MYND4
TCGA: The Cancer Genome Atlas
TERTp: telomerase reverse transcriptase promotor
TMB: tumor mutational burden
TMZ: temozolomide
WES: whole‐exome sequencing
WGS: whole‐genome sequencing
WHO: World Health Organization

## 1 Introduction

Glioblastoma (GBM) is an incurable brain cancer with an incidence of 3.2/100 000 [1]. Biomarker‐driven targeted therapy has proven effective in many cancer types and seems promising in GBM based on case stories with specific aberrations [2, 3, 4]. This includes gene fusions that have resulted in approval of tropomyosin receptor kinase (TRK) inhibitors for TRK fusion‐positive cancers, regardless of histology [5, 6]. With the comprehensive genomic characterization of GBM in 2008 [7] and the revised World Health Organization (WHO) classification of brain tumors in 2016 with integration of molecular analyses, the hope was that it would contribute to better treatment options in GBM. Genomic testing is being used in the clinic today [8, 9] but unfortunately has not yet translated into a better overall survival (OS). Standard 1st‐line treatment remains concurrent radiotherapy (RT)/temozolomide (TMZ) followed by adjuvant TMZ with a median progression‐free survival (PFS) of 7 months and a median OS of 14–22 months, depending on prognostic and predictive markers like isocitrate dehydrogenase (*IDH*) status and O6‐methyl‐guanine‐DNA‐methyl‐transferase (MGMT) promotor status [10, 11, 12]. Some explanations for lack of clinical impact of the genomic analysis into a better OS might be that majority of samples in international databases represent both primary and relapse samples, can have unknown *IDH* and/or MGMT status, and have limited information of treatment exposure. The latter can change the genetic composition with possible development of hypermutated phenotypes [13] or higher chromosomal instability (CI) [14]. Also, overrepresentation from specific demographic areas can cause challenges for data evaluation as different ethnic groups can have a heterogeneous genetic composition [15]. Lastly, at initiation of international databases such as the The Cancer Genome Atlas (TCGA), molecular diagnostics was not incorporated to the same extent as today and retrospective work with methylation profiling on cases from the databases has shown 12% of samples with discrepancies, which consequently could have a new diagnosis assigned [16]. To face some of these challenges, we have performed a prospective study with inclusion in the Copenhagen GBM cohort (CGC) to determine the genomic profile in newly diagnosed patients with GBM, with the purpose to investigate whether a genomic profile could potentially lead to an altered treatment strategy in both 1st‐ and 2nd‐line treatment and to investigate prognostic/predictive relevance of genomic variants. Evaluation of inclusion in clinical trials for the individual patient was investigated in the relapse setting only since we did not have approval for experimental treatment in the 1^st^ line at our institution. To our knowledge, this is the first study performed after the 2016 WHO classification with prospective translational results, including clinical, pathological, and genomic data on all included patients.

## 2 Materials and methods

### 2.1 Collection of tissue

**Fig. 1:**
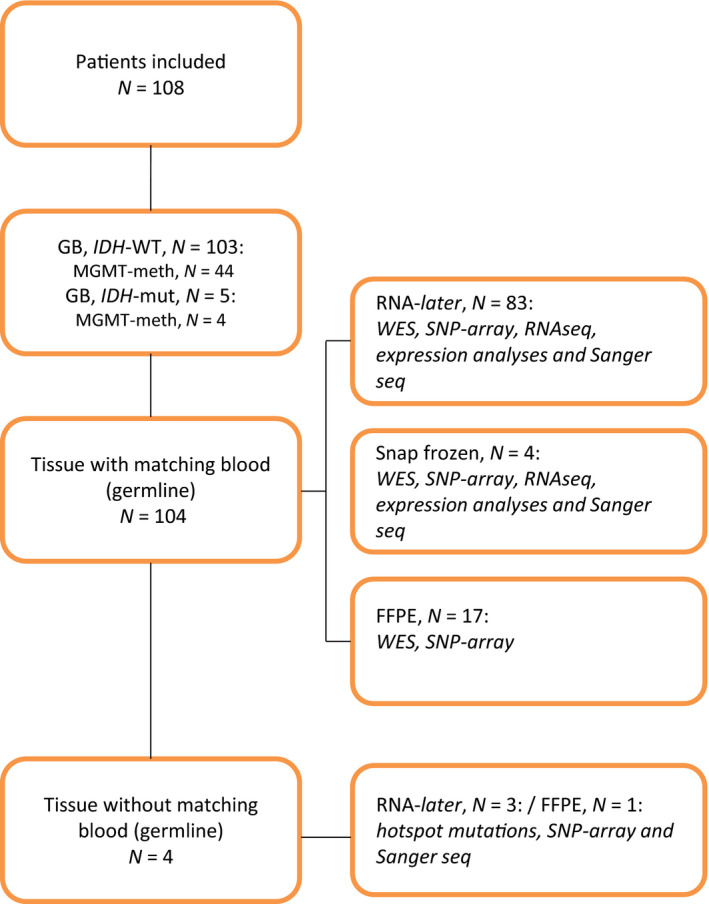
CONSORT diagram. The inclusion criteria were as follows: newly diagnosed GBM, no previous treatment from a lower grade glioma, signed informed consent. Diagnosis based upon WHO classification for brain cancers 2016. DNA was used to perform WES, SNP array, and Sanger seq, and RNA was used to perform RNA‐seq, expression, and fusion analyses. Only tissue preserved in RNA‐later or as snap‐frozen could be used to determine TERTp status and subtype division. meth; methylated; mut: mutated; WT, wild‐type.

Over a 2½‐year period from February 2016 to August 2018, we included 108 patients with newly diagnosed GBM at Rigshospitalet, Copenhagen. The diagnosis was based on the WHO classification from 2016 with histopathology and molecular examination for *IDH* and MGMT status [11]. Patients, who had previously received treatment for a lower grade glioma with transformation into a grade IV GBM, were not included. In the first year, we included all newly diagnosed patients, but shifted to include only patients suitable for RT/TMZ due to a potential clinical impact on future treatment. All patients gave informed, signed consent prior to surgery. Whenever possible, 5‐ALA was used during surgery [17]. Three representative tissue specimens from diagnostic surgery were immediately preserved in RNA‐later for optimal DNA and RNA quality. In case of insufficient amount of tumor material, we used formalin‐fixed paraffin‐embedded (FFPE) tissue or snap‐frozen tissue. Blood sample (10 mL) was taken to filter for germline variations (Fig. 1). The project was carried out in accordance with the Declaration of Helsinki and with approval from the National Danish Ethics Committee (Journal number: H‐3‐2009‐136 and 1707335) and Danish Data Protection Agency (Journal numbers: 2014‐41‐2857 and VD‐2018‐204 with I‐suite number: 6447).

### 2.2 Clinical data

Clinical data were noted from patient interviews and medical records, including age at diagnosis, location of the tumor, extent of surgery, performance status (PS) and corticosteroid dose before oncologic treatment, treatment given, number of cycles of adjuvant TMZ completed, completed full planned treatment yes/no, relapse surgery yes/no, PFS, and OS. Date of datalock was 10.01.2019.

### 2.3 Pathological examination

Every sample underwent standard pathological examination with immunohistochemistry for GFAP, map2, Olig2, *IDH*, p53, *ATRX,* and Ki67 index. For patients < 55 years with normal *IDH* status, sequencing of codon 132, 140 and 172 was done. MGMT status was determined by PCR with pyrosequencing of four CpG sites in the promotor region of MGMT, using the therascreen MGMT system (Qiagen, Hilden, Germany) on bisulfite‐treated genomic DNA. The cutoff value was 10%. When in doubt of diagnosis, 850K methylation with infinium methylation EPIC BeadChip array which targets > 850 000 methylation positions in the human genome was performed. In young patients and/or midline tumors and/or *IDH*‐WT in combination with *ATRX* loss, an analysis for H3K27M was added with sequencing of *H3F3A* codons 28–35 with a sensitivity of 20% tumor cells.

### 2.4 Whole‐exome sequencing

Whole‐exome sequencing (WES) was performed using DNA from tissue and blood. DNA from tumor samples (tDNA) was extracted using the AllPrep DNA/RNA purification kit and the QIACube workstation (Qiagen) according to manufacturer's instructions. Genomic DNA from whole blood samples (gDNA) was isolated using the liquid handling automated station (Tecan, Männedorf, Switzerland). Purified DNA was quantified using the Qubit instrument (Life Technologies, Thermo Fisher Scientific, Waltham, MA, USA). Both tDNA and gDNA (200 ng) were fragmented to approximately 300 bp using Covaris S2 (Agilent, Santa Clara, CA, USA), and adaptor ligation was performed using KAPA HTP Library Preparation Kit (Roche, Basel, Switzerland). Exomes were enriched with SureSelectXT Clinical Research Exome kit (Agilent). Paired‐end sequencing (2 × 100 bp or 2 × 150 bp) was performed to gain an average coverage of 50–100×, using the HiSeq2500 or NextSeq500 platforms from Illumina (San Diego, CA, USA). Raw sequencing data were processed using CASAVA‐1.8.2 [18]. Reads were aligned to the human reference genome (hg19/GRCh37) using CLC Biomedical Genomics Workbench (Qiagen), and variant calling was performed above 10% frequency in the tumor DNA. Somatic variants were identified by excluding variants found in blood WES data from the patient, and further analyzed using ingenuity variant analysis (Qiagen). A gene list based upon frequent mutated genes in GBM was used to filtrate for mutation calling in ingenuity (Table S1), and mutations were categorized based on the likelihood of being pathogenic [19]. *SMYDA* and growth factor receptor bound (*GRB2*) were further investigated with survival data from the GBM dataset of TCGA using the R package ‘RTCGA’ [20]. For each of the investigated genes, a given sample was considered as being a positive case for the respective gene, if any mutation was observed for this sample. A given sample could occur as a positive case for more than one gene. Kaplan–Meier survival curves were fitted to the survival data using the r package ‘survival’ [21]. The survival curves were plotted using the r package ‘survminer’ [22], and the significance of the observed differences in survival was based on the log‐rank method.

### 2.5 Tumor mutational burden

Paired‐end sequencing reads with a length of 150 bp were aligned against the GRCh37.p13 reference genome using BWA‐MEM 0.7.15. Somatic variants were called using Mutect2 according to the GATK best practices for somatic short variant discovery using GATK 4.0.10.1. Variants filtered by Mutect2 and variants annotated with an allele frequency > 5% in gnomAD were excluded from the call set. The variants were further hard filtered by only including single nucleotide variation and INDELs in coding regions. Finally, variants called at sites with a coverage of < 10× and an allele depth of < 5× were excluded. The tumor mutation burden was calculated as the number of nonfiltered variants divided by the number of bases with a coverage of > 10× in all coding regions of the genome. Tumor mutational burden (TMB) estimates were reported as mutations per megabase (Mb).

### 2.6 Analysis of somatic copy number alterations

CytoScan assay (Affymetrix, Santa Clara, CA, USA) was performed on tumor samples according to the manufacturer's instructions. OncoScan assay (Affymetrix) for analysis of FFPE material was performed according to the manufacturer's instructions. OSCHP files from OncoScan and .CEL files from the CytoScan assay were imported into NEXUS v8.0 (BioDiscovery, ElSegundo, CA, USA) and used for the analysis and visualization of somatic copy number alterations (SCNA)s and loss of heterozygosity (LOH). SCNAs (loss, gain, biallelic loss, or high amplification) and LOH calls for each sample were confirmed by visual inspection and followed by manual interpretation of whole‐exome profiles. Tumors were assessed for CI. CI was assigned if the sample displayed more than 15 SCNAs; that is, segmental chromosomal aberrations (SCA) and/or numerical aberrations.

### 2.7 Gene expression analysis

RNA was reverse‐transcribed and used for cRNA synthesis, labeling, and hybridization with GeneChip® Human Genome U133 Plus 2.0 Array (Affymetrix) according to the manufacturer's protocol. The arrays were washed and stained with phycoerythrin‐conjugated streptavidin using the Affymetrix Fluidics Station 450, and the arrays were scanned in the Affymetrix GeneArray 3000 7G scanner to generate fluorescent images [23]. Cell intensity files (.CEL files) were generated in the GeneChip Command Console Software (AGCC; Affymetrix). Cell files were preprocessed using the robust multichip average (RMA) method [24, 25, 26]. Following normalization, the data were visualized and analyzed using the qlucore omics explorer software (Qlucore, Lund, Sweden). Gene set enrichment analysis (GSEA) was performed as described [27] All transcripts were included in the analysis and matched toward the entire mSigdb (https://www.gsea‐msigdb.org/gsea/index.jsp) or the HALMARK gene sets as indicated. We employed microarray since RNA sequencing (RNA‐seq) was not available for all samples.

### 2.8 Fusion analysis

RNA sequencing was done using the TruSeq Stranded Total RNA Library Prep Kit and was sequenced on the NextSeq500 (Illumina). Raw sequencing data from the Illumina sequencing platforms were processed with CASAVA‐1.8.2. FusionMap bioinformatics tool (Array Suite, ThermoFisher) was used for screening of fusion transcripts as previously published [28].

### 2.9 Determining of TERTp

Telomerase reverse transcriptase promotor region (TERTp) mutation was determined using Sanger sequencing for the two most common mutations; c.‐124C>T and c.‐146C>T. In brief, primers were designed to produce PCR products covering the sites. The purified PCR products were sequenced by Sanger sequencing using an ABI 3730 DNA Analyzer according to the manufacturer's instructions (Applied Biosystems, Foster City, CA, USA).

### 2.10 Subclass analysis

An in‐house developed classifier based on the study data (E‐GEOD‐68850) [29] was used to assign the tumor into one of the three subtypes of interest (classical, mesenchymal, and proneural). Briefly, the raw intensity .CEL files were preprocessed by quantile normalization and gene summaries were extracted *via* RMA. The expression values of 4324 classifier genes were standardized across samples. The 2‐dimensional t‐distributed stochastic neighbor embedding (t‐SNE) algorithm was applied to a fraction of the dataset multiple times. A sample was considered to belong to a subtype when its corresponding Gaussian model gave the maximum probability density among the rest of the models and that probability was > 0.001. Since subclass division was based upon expression analysis from snap‐frozen tissue or tissue in RNA‐later, FFPE samples were noted N/A.

### 2.11 Statistics

Overall survival and PFS were estimated using the Kaplan–Meier method. Comparison of selected genes with SCNA's (biallelic loss, amplification, LOH, deletion, and LOH) and clinical characteristics, including comparison of selected genes with biallelic loss or amplification and completing RT/TMZ, was calculated using Fisher's exact test. For univariate and multivariate analyses and OS, we used the Cox proportional hazards model and results were presented as hazard ratios (HR) with 95% confidence interval (CI). *P*‐values < 0.05 were considered significant. Statistical analyses were done using spss (v.25.0; IBM, Armonk, NY, USA) and rstudio (v.3.5.2; RStudio, Boston, MA, USA).

## 3 Results

### 3.1 Patient characteristics

A total of 108 patients were included (Table 1). The patients resembled a standard clinical setting with patients eligible for RT/TMZ. *ATRX* mutation was found in five patients (4.6%), four of these (3.7%) were under the age of 45, and three (2.8%) had an *IDH* mutation. Median PFS and OS were 7.8 and 16.3 months, respectively.

**Table 1:** Patient characteristics. PS and corticosteroid dose were noted approximately 1 month after surgery when the patient was seen at Department of Oncology, before start on oncologic treatment. adj: adjuvant.

Number of patients	108
Sex	
Female (%)	44 (41)
Male (%)	64 (59)
Age at diagnosis, median (range)	62 (18–89)
PS, median (%)	0 (0–4)
0	59 (55)
1	34 (32)
2	13 (12)
3	1 (1)
4	1 (1)
MGMT‐methylated (%)	48 (44.4)
*IDH* wild‐type (%)	103 (95)
*ATRX* mutated (%)	5 (4.6)
Corticosteroid dose, mg (median, min‐max)	15 (0–75)
Treatment (%)	
RT/TMZ and adj TMZ	83 (77)
RT/TMZ plus IT or placebo (trial)	6 (6)
IT/RT and adj IT (trial)	4 (4)
TMZ monotherapy	2 (2)
60 Gy/30F	5 (5)
34 Gy/10F	7 (7)
None	1 (1)
RT/TMZ and adj TMZ completed (%)	31 (37)
Median number of cycles (range)	5 (0–11)
Still on‐treatment at datalock (%)	6 (7)
Relapse surgery (%)	
Yes	43 (40)
No	41 (38)
Not yet progressed	24 (22)
Tumor location (%)/ complete resection (%)	
Frontal	33 (31)/ (76)
Parietal	22 (20)/ (91)
Temporal	30 (28)/ (60)
Occipital	8 (7)/ (100)
Brainstem	1 (9)/ (0)
Other^a^	14 (13)/ (64)
PFS, median (months)	7.8
MGMT‐unmethylated	6.7
MGMT‐methylated	13.7
OS, median (months)	16.3
MGMT‐unmethylated	14.7
MGMT‐methylated	Not reached

^a^ Tumor overlapping two lobes.

### 3.2 The genomic landscape, TERT promotor status, and fusion analyses

**Fig. 2:**
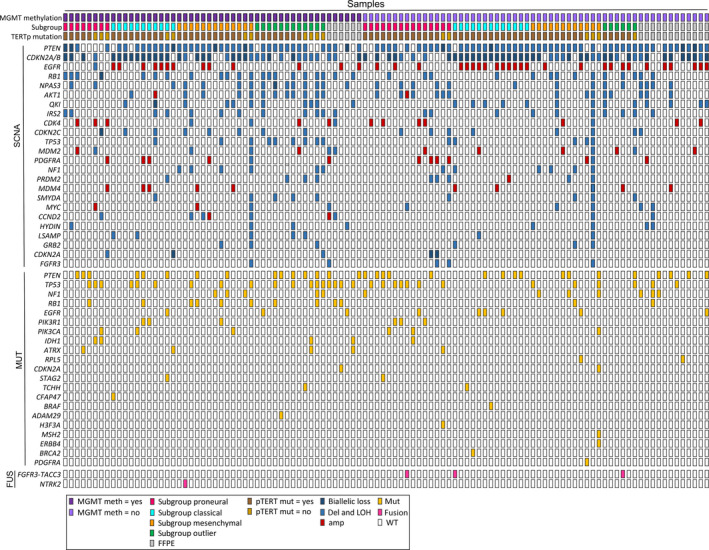
Landscape of SCNA in selected genes altered ≥ 5 patients, GBM‐specific mutations and fusions listed hierarchically. *N* = 108. Only mutations categorized as pathogenic are shown. The most frequently aberrated genes were PTEN, CDKN2A/B, EGFR, RB1, and NPAS3, and the most frequent mutations were in PTEN, TP53, NF1, RB1, and EGFR. Abbreviations: mut: mutated; fus: fusions.

Single nucleotide polymorphism (SNP) array was successfully performed in all samples and WES in 104 (96.3%) samples, where both tumor and blood samples were available. Figure 2 presents the genomic landscape of SCNAs present in ≥ 5 patients, every GBM‐related mutation with pathological significance and the identified gene fusions. The top five most aberrated genes were *PTEN, CDKN2A/B*, epithelial growth factor receptor (*EGFR*), *RB1*, and *NPAS3*. The most frequent mutations were in *PTEN, TP53, NF1, RB1,* and *EGFR*. A plot of genome‐wide copy number changes is shown in Fig. S1, and a list of all identified mutations is shown in Table S2. TERTp was mutated in 74 (68.5%) of the samples with 51 (68.9%) having the c.124 C>T mutation and 23 (21.3%) having the c.146 C>T mutation, respectively. In the 17 patients with FFPE material, TERTp status was not assigned. Mutations in TERTp did not relate to worse median OS (data not shown). We investigated all patients for fusions with *FGFR*, neurotrophin tyrosine receptor kinase (*NTRK*), and *MET* and identified *NTRK2* in one patient with a MGMT‐methylated tumor and *FGFR3‐TACC3* in 3 patients (2.8%) all of which were in MGMT‐unmethylated tumors.

### 3.3 Subtype division

Subtype division was possible in 89 patients (82.4%) and was equally distributed with 21 (23.6%) having proneural, 26 (29.2%) classical, and 25 (28.1%) mesenchymal subtype. Seventeen (19.1%) patients were outliers. We did not find subgroup division to be prognostic for median OS, nor predictive of response to RT/TMZ (Fig. S2A,B). After adjusting for MGMT status and regardless of treatment, the classical subgroup and the outliers group had a significant difference in median OS and the proneural group a borderline significant difference in median OS depending on the status of MGMT. However, numbers are small, and the median OS was not reached in the proneural and the classical subgroup at time of datalock (Fig. S3A–D).

## 4 Individualized treatment

Each genomic report was discussed at biweekly tumor board meetings with specialists from molecular biology, clinical genetics, bioinformatics, pathology, and medical oncology. It was feasible to have the results ready for time at first progression. In the study period, we identified one patient with *NTRK2* fusion and several patients with *IDH* mutation eligible for experimental treatment based on the on‐site available trials. One patient with NTRK2 fusion was included in the NAVIGATE trial (EudraCT: 2015‐003582‐28), and one patient with *H3F3A* mutation was included in the international ONC‐201 protocol (*NCT03295396*). Other potential targets were mutations in *EGFR, CDK4/6, NF1, FGFR3*, and *FGFR3‐TACC3* fusions.

### 4.1 Genomic changes and OS

We further investigated genomic alterations and OS. First, we tested clinical variables in a univariate analysis and found age < 70 years, PS 0–1, and corticosteroid dose < 10 mg once daily to be statistically correlated with better survival. MGMT status and genes with SCNAs in ≥ 5 patients were also tested in a univariate analysis. MGMT unmethylation, alterations in *GRB2*—and SET and MYND4 (*SMYD4*) genes were significantly correlated with worse survival (Table S3). We tested the prognostic value in the TCGA dataset and found a trend toward a worse median OS in the *GRB2*‐mutated samples and no correlation in the *SMYD4* mutated samples (Fig. S4). However, numbers are small as *GRB2* and *SMYD4* were present in only 5/596 and 6/596 samples, respectively.

### 4.2 Testing for genomic variations with correlation to treatment completion

A total of 83 (76.9%) patients were eligible for and received RT/TMZ. 31 patients (37.4%) completed the planned treatment, and 46 patients (55.4%) did not. Six patients (7.2%) were still on‐treatment at time of datalock and were excluded in the following analysis. The main reason for not completing the planned treatment was progression. Patients completing the planned treatment had a statistically significant median survival benefit of 25.6 vs. 14.6 months for patients not completing the treatment (*P* < 0.000) even though all patients were eligible for concurrent treatment upfront and hence should be comparable at start of treatment. The known predictive value of MGMT status with TMZ treatment was confirmed in our dataset as patients with MGMT‐methylated tumors had a median survival of 29.6 vs. 15.4 months in patients with MGMT‐unmethylated tumors, *P* = 0.001 (Data not shown). After adjusting for the three clinical (age, PS, and corticosteroid dose) and genetic variables (MGMT, *GRB2*, and *SMYDA*), the result was still significant, showing that completion of therapy was not alone dependent of belonging to a good prognostic group (data not shown). Next, we investigated the predictive potential for completing the treatment by testing genes with amplification and/or biallelic loss and the three clinical variables but could not identify any besides the known MGMT status (*P* = 0.02; Table S4).

### 4.3 Tumor mutational burden

**Fig. 3:**
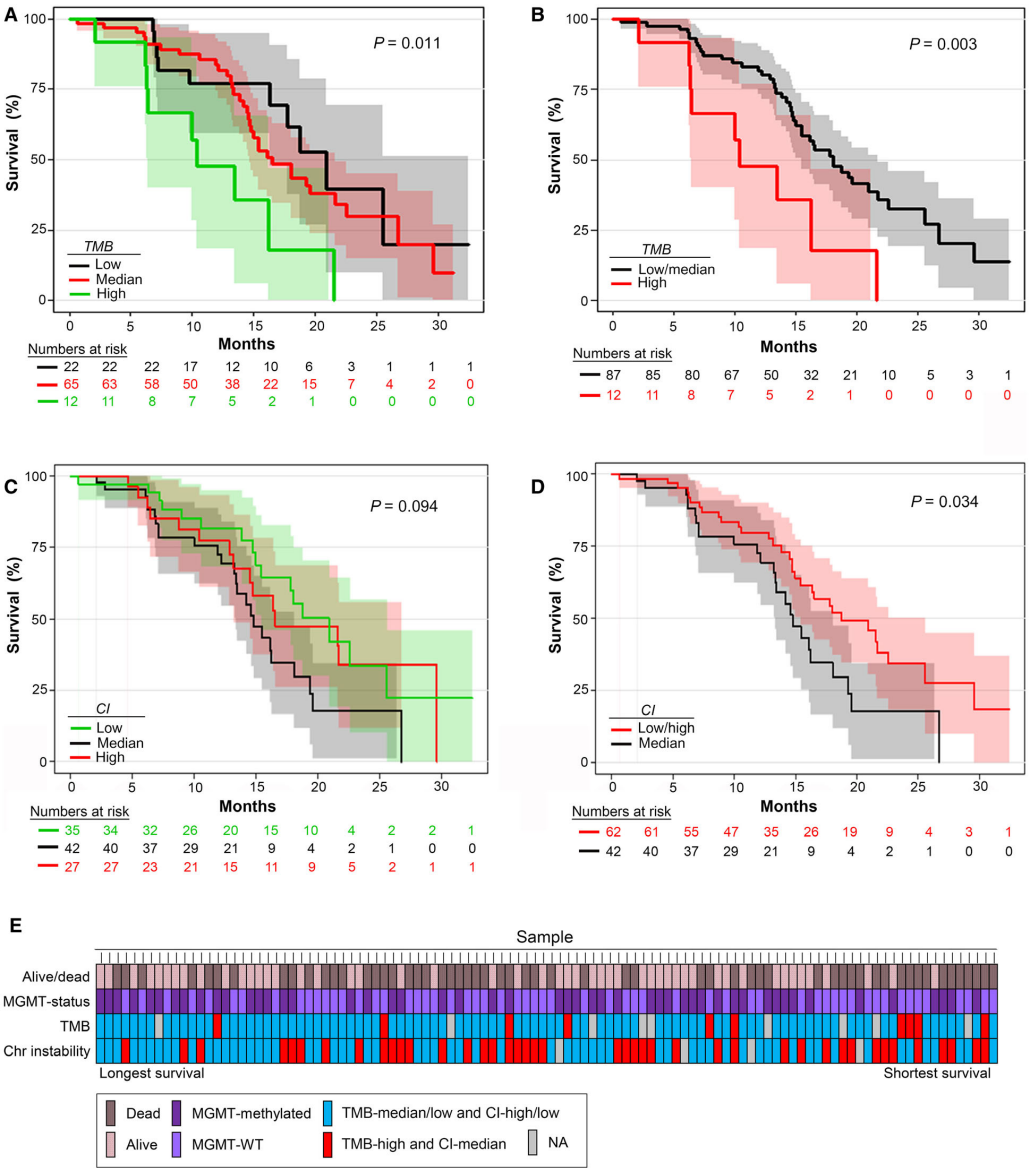
Kaplan–Meier curves with numbers at risk for OS for (A) TMB‐high, TMB‐median and TMB‐low, (B) TMB‐high and TMB‐median/low, (C) CI‐high, CI‐median, and CI‐low, and (D) CI‐median and CI‐high/low. (A, B) TMB was defined as number of mutations/Mb into low (0–1.5, *N* = 22), median (1.6–2.9, *N* = 65), and high (≥ 3.0, *N* = 12). Total *N* = 99. (A) TMB‐high vs. TMB‐median and low had a significantly worse median OS of 10.4 months (95% CI: 5.7–15.1) vs. 16.5 months (95% CI: 13.4–19.7) and 20.9 months (95% CI: 16.7–25.1), respectively (*P* = 0.011). (B) Groups were segregated into TMB‐high (*N* = 10) vs. TMB‐low/median (*N* = 89). A statistically significant difference remained with median OS of 18.0 months (95% CI: 14.8–21.2) in the combined group (*P* = 0.003) and with a HR calculated using a Cox regression analyses of 0.29 (95% CI: 0.14–0.61, *P* = 0.001) in TMB‐median/low vs. TMB‐high, respectively. (C, D) CI was split into low (0–7 SCA, *N* = 35), median (8–15 SCA, *N* = 42), and high (> 15 SCA or aneuploid background, *N* = 27). Total *N* = 104. (C) CI‐median vs. CI‐high and CI‐low had a worse median OS of 14.8 months (95% CI: 21.5–17.1) vs. 16.5 months (95% CI: 8.1–24.9) and 20.9 months (95% CI: 16.0–25.8), respectively. Results were borderline significant (*P* = 0.094). (D) Groups were then segregated into CI‐median vs. CI‐high/low with a median OS of 18.7 months in the combined group (95% CI: 13.8–23.7; *P* = 0.034). (E) All patients and TMB, CI, and MGMT status, ranged with highest survival first.

The evaluation of TMB was feasible in 99 patients (91.7%). Median TMB before diagnosis was 2.2/Mb with a range of 0.7–7.1 and one extreme outlier of 36.2. When dividing TMB into low (0–1.5, *N* = 22), median (1.6–2.9, *N* = 65), and high (≥ 3.0, *N* = 12), we found a worse survival of 10.4 months in the TMB‐high patients vs. 16.5 and 20.9 months in the TMB‐median and TMB‐low, respectively (*P* = 0.011). We then merged TMB‐median and low and compared them to TMB‐high tumors, still yielding statistically significant results in median OS with 18.0 months in the combined group (*P* = 0.003) and with a HR of 2.87 (95% CI: 1.38–5.97) in TMB‐high vs. TMB‐median/low, respectively (*P* = 0.005; Fig. 3A,B). After testing in a multivariate analysis with adjustment for the above identified three clinical variables (age, PS, and corticosteroid dose) and MGMT status, the results remained significant with *P* = 0.009, HR: 3.29 (95% CI: 1.35–8.02; Table S5A,B). Results were, however, not confirmed in an independent validation dataset (TCGA; filtered for age 30–80 years and survival > 90 days from diagnosis).

### 4.4 Chromosomal instability

Next, we explored whether CI could prognosticate OS based on number of SCNAs and/or aneuploid background. Evaluation of CI was possible in 104 patients (96.3%). CI was divided into low (0–7 SCA, *N* = 35), median (8–15 SCA, *N* = 42), and high (> 15 SCA or aneuploid background, *N* = 27). CI‐median had the worst median survival of 14.8 months vs. 16.5 and 20.9 months in CI‐high and CI‐low, respectively, (*P* = 0.094). When merging the two groups with the better survival, a median survival was 18.7 months (*P* = 0.034) and HR of 1.78 (95% CI: 1.04–3.04) in CI‐median vs. CI‐high/low, respectively (*P* = 0.037; Fig. 3C,D). When adjusting for MGMT status and the three clinical variables (i.e., age, PS, and corticosteroid dose), the difference was still borderline significant (*P* = 0.13), HR: 1.51 (95% CI: 0.88–2.60; Table S5A,B). We then compared the less favorable groups (TMB‐high/CI‐median) to the favorable group (TMB‐median/low + CI‐high/low) and found a difference in median survival of 14.8 months (95% CI: 12.4–17.1) vs. 20.9 months (95% CI: 15.9–25.9), respectively (*P* = 0.008; Fig. S5). With the new WHO diagnostic criteria, GBM, *IDH*‐mutated is defined as an independent diagnosis with a better prognosis than GBM, *IDH*‐WT. Therefore, we performed the same analyses with survival and TMB and CI, respectively, with exclusion of *IDH*‐mutated samples. The results did not change our interpretation of the data. An overview of survival, MGMT status, TMB, and CI is shown in Fig. 3E, and a histogram distribution of TMB and CI is shown in Fig. S6.

### 4.5 Gene expression in subgroups, CI and TMB categories

**Fig. 4:**
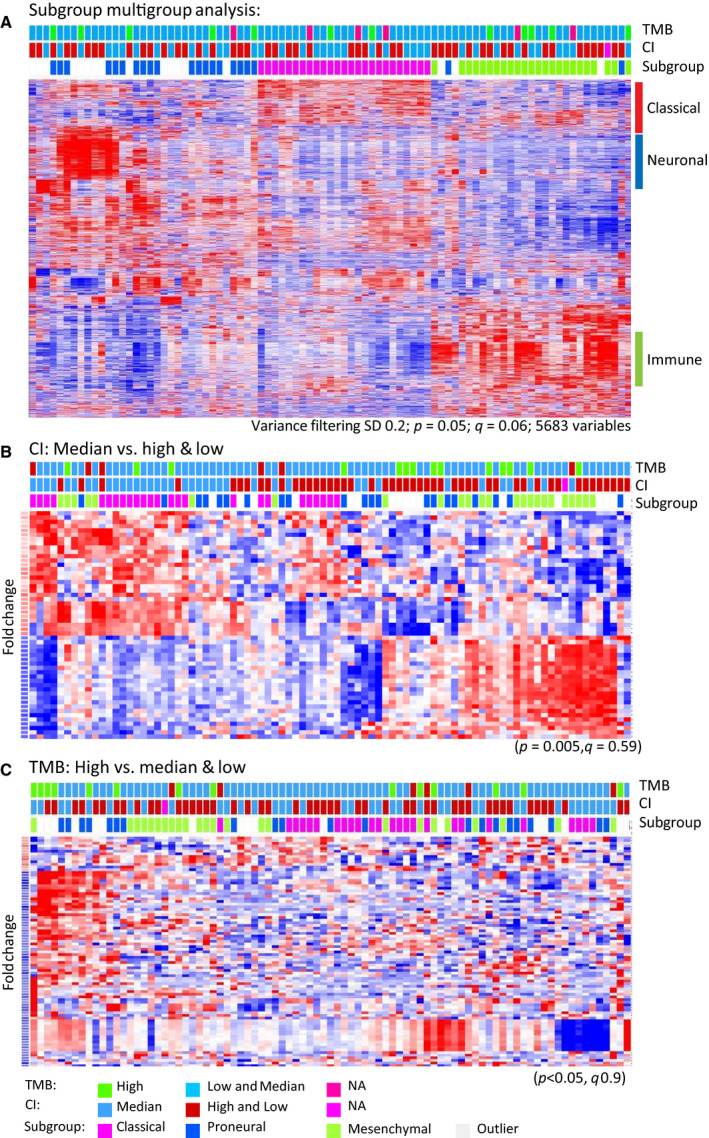
(A) Two‐way hierarchical cluster of GBM tumor classes following variance filtering. Tumor classes are indicated on top of the heat map together with the CI and TMB categories. Category labels and statistics are indicated below the heat map. (B, C) Two‐way comparisons of CI‐median versus high/low categories and TMB‐high versus median/low categories, respectively. As described for panel (A), categories are indicated on the top of each panel.

Gene expression profiling was feasible in 88 samples and was performed to search for possible biomarkers associated with reduced survival in the CI and TMB groups, respectively. One sample from FFPE tissue and one outlier was omitted from the analysis, so in total 86 samples, consisting of 25 classical, 24 mesenchymal, 20 proneural, and 17 outliers, were included in the analysis. Figure 4A depicts a two‐way hierarchical clustering of the subclasses with 5683 variables (*P* = 0.05; SD = 0.2). Variables associated with neuronal tissue and infiltrating immune cells were apparent in the proneural and mesenchymal tumors as well as transcripts defining the classical subtype. Outliers clustered mainly among the proneural GBMs, indicating that they share many features and are likely to represent proneural subtypes with embedded normal tissue as described previously [30]. With exception of the mesenchymal tumors that exhibited more than 2000 differentially mRNAs, the differences between the subgroups were moderate with only 600–900 differentially expressed mRNAs. In agreement with the similarity to the proneural GBMs, outliers were only distinguished by 119 transcripts.

Since patients belonging to the TMB‐high and CI‐median groups had reduced survival, we compared these groups with the remaining samples. As shown in Fig. 4, the TMB and CI samples were distributed over all subgroups in agreement with the finding that subgroups were not significantly associated with survival. We performed a two‐way comparison of the CI‐median and TMB‐high category against the remaining categories (Fig. 4, panels B,C). As revealed by the modest *p* and *q* values, we found only minor differences between the categories—particularly the TMB‐high group was very similar to the median and low groups. In the TMB‐high category, upregulated mRNAs were found in a subset of the samples. Noteworthy, a number of *DLX* genes (*DLX1, DLX2,* and *DLX5*; Tables S6 and S7) previously described in GBM prognosis [31] were upregulated. Median CI was associated with upregulation of 34 transcripts. The most upregulated mRNAs, including *SLITRK3,* are listed (Tables S6 and S7). Since the number of differentially expressed genes was modest, we supplemented with a GSEA employing the molecular signature database HALLMARK sets to depict perturbed molecular pathways (Tables S6 and S7). CI‐median tumors exhibited enrichment of genes involved in cholesterol homeostasis and WNT signaling. The GSEA moreover showed a tendency in the TMB‐high group toward enrichment of the transforming growth factor beta (TGF beta) and hedgehog pathways that previously have shown to affect OS and growth of tumors by involvement in many cellular processes, for example, gene expression, cell differentiation, and growth.

## 5 Discussion

Here, we report a prospective study using the CGC with treatment‐naïve GBM patients. Findings represented SCNAs, mutations, and clinical variables and new findings were worse survival in patients with deletion and/or LOH of *GRB2* and *SMYD4,* both genes located in chromosome 17. We identified a higher prevalence of mutation in the two genes as compared to the TCGA dataset. A trend toward a worse median OS in the *GRB2*‐mutated samples was found (Fig. S4). This potential prognostic mutation has not been reported previously and should be evaluated in future studies. The functional GRB2 protein acts as a negative regulator of the RAS pathway by inhibiting EGFRs [32], and *SMYD4* is involved in inhibition of gene expression and has been suggested as a tumor suppressor gene [33, 34]. In 4/6 patients with *GRB2* alterations, alterations in *SMYD4* were also present. To our knowledge, no known interaction between the two genes exists. All samples but two had a SCNA in either *PTEN* and/or *CDKN2A/B*, essential for the development of GBM. The obvious targeted treatment would be a tyrosine kinase inhibitor. However, until now all late phase trials of targeted therapies in GBM have been unsuccessful [35]. In our study, sequencing depth was sufficient to find possible drivers, but sampling bias can pose another challenge due to intratumor heterogeneity and undetectable, targetable subclones can be present at diagnosis [36]. In some cases, it might be worth considering a deeper sequencing for selected targetable oncogenic drivers like *BRAF* in histologic subtypes [37] or NTRK fusions in *IDH*‐WT patients [38].

### 5.1 Subclass division did not predict outcome

To investigate another approach of predictive value, we divided our cohort into the three subclasses: (a) proneural; (b) classical, and (c) mesenchymal according to Verhaak *et al*. [30]. However, we failed to validate their predictive value in our cohort. Recent work from TCGA showed that the predictive advantage to TMZ in the classical subtype and the prognostic value in the proneural subtype was attributed to MGMT methylation [39], why we investigated each subclass stratified for MGMT status. The known predictive advantage of MGMT methylation was confirmed, and a borderline significant difference in median OS was found in the classical subgroup and the outliers. This lack of coherence was also found in a study by the German Glioma Network [40]. In agreement with the molecular classification based on the classification scheme generated by Verhaak *et al*. [30], the GSEA of the subgroups showed that gene sets, for example, classical, proneural, and mesenchymal were enriched. The outliers seem to belong to the proneural subgroup. The mesenchymal subgroup had more differentially expressed genes and might represent an even more heterogeneous tumor as compared to the other subgroups. However, numbers are small and should be interpreted accordingly.

### 5.2 Treatment completion and genetic composition

Not surprisingly, we found a statistical survival benefit in the group of patients able to complete the planned concurrent treatment, even after stratifying for the three clinical and genetic variables. Indisputable, it would be extremely valuable if we could predict who would benefit from RT/TMZ to select future patients for first‐line standard treatment or first‐line experimental treatment. Unfortunately, we could not find a predictive genetic composition for completing RT/TMZ and conclude that more research is needed in this important question. One approach might be to perform hierarchical clustering of expression data in the two groups or to investigate TMB and CI in a larger cohort. It is worth noticing that only a small subset of the intended/scheduled treatment was completed in 31 (40.3%) out of 77 evaluable patients with a median number of five cycles completed (range 0–11). A full course with RT/TMZ and adjuvant TMZ includes > 8 months of treatment, and with a median PFS of 7.8 months, a large percentage of patients will not be able to complete the full treatment.

### 5.3 TMB and CI predicts outcome

No cutoff values or standardized analyses exist for TMB or CI in GBM [41]. Estimation of the two can be influenced by many factors that can hamper comparison between studies, for example, sequencing technology and depth, data processing, preservation of samples, or previous treatment. Therefore, we defined our study‐specific cutoff values, as described in section 1.4.3, based on the literature with TMB ranging between 1 and 3 mutations/Mb [42, 43, 44]. The median TMB in our dataset was 2.2 mutations/Mb with one extreme outlier of 36.2 mutations/Mb. Patients with TMB‐high had a significantly worse survival as compared to both median and low TMB, respectively. The correlation with TMB‐high and worse OS has been found in other brain tumors [45], but most studies have been performed in immunotherapy (IT)‐treated patients only and with a focus on the predictive potential of TMB to IT. Here, we find that TMB might be used as a stratification factor for selection of patients to experimental treatment, including other therapies than IT. The expression of mRNAs showed no great difference in the TMB groups, but we identified upregulation of *EBF1* and growth arrest‐specific 2 (*GAS2*) along with *DLX* genes in the TMB‐high group. *EBF1* encodes for transcription factors, and *GAS2* is involved in apoptosis. The *DLX* genes inhibit several cytokine‐signaling pathways [32, 46]. GBM is often categorized as a ‘cold’ tumor with a less active immune system, but a TMB‐high tumor can have a high immunological activity due to the high load of neoantigens and these factors combined might explain the higher aggressiveness in this subgroup. TMB has proved to be a useful clinical marker for immune therapy in other cancer types [42, 47, 48, 49, 50, 51] and is being used as a marker for experimental treatment in GBM [52] but until now, has not proven effective in GBM [53, 54, 55]. As shown in our results, TMB might have a role in future treatment stratification, indicating a need for a more aggressive strategy for the TMB‐high group. In addition, TMB combined with CI showed even stronger biomarker potential (Fig. S5). The combination has been investigated by others [56] but to our knowledge, only in the setting of biomarker potential to IT. CI is a shared feature across 60–80% of cancer histologies [57] and can cause inflammation, activation of the innate immune system, universal hypomethylation with general activation of genes, and a deficient mismatch repair (MMR) system [58, 59]. In concordance with these observations, we found upregulation of SLIT and NTRK‐like (*SLITRK3*), T‐cell receptor delta constant (*TRDC*), and vascular endothelial growth factor‐A in the CI‐median group. *SLITRK3* has previously been identified in tumors and *TRDC* participates in recognition of antigens [32, 60]. The VEGF‐family is highly expressed on the surface of GBM cells and is involved in development of resistance to Bevacizumab [61]. High and low CI can both show slow tumor growth, high due to the enormous DNA instability causing an unstable cancer cell not able to perform its malignant potential, and low due to the slow growth [62]. However, optimal CI can create equilibrium between genomic chaos and cell survival and can drive tumor heterogeneity and treatment resistance, causing a highly aggressive tumor. This was defined as CI‐median in our cohort and lead to significantly worse survival compared to the CI‐high and CI‐low group. To our knowledge, this has not been shown before and needs further investigation. When we combined the less favorable group (TMB‐high and/or CI‐median) vs. the favorable group (TMB‐median/low plus CI‐high/low), the two clustered groups showed significant differences in median OS, indicating the great potential of clinical application of TMB and CI. Unfortunately, we could not validate our TMB findings in a TCGA cohort of 40 samples. Some explanations might be the missing information of newly diagnosed samples vs. treatment exposed samples that can dilute results and the question of cutoff values. In our TMB‐high group, 12 patients were identified and in the TCGA validation set, only four patients were categorized as TMB‐high when using our study‐specific cutoff values. Therefore, the predictive value of TMB and CI should be tested in a larger cohort, preferably in samples diagnosed by the 2016 WHO diagnostic criteria and with standardized methods.

### 5.4 Impact of molecular profiling on GBM treatment

The hopes of extensive molecular profiling are in finding targeted, efficient treatment. We identified potentially targetable aberrations including gene fusions in *NTRK2* and *FGFR3‐TACC3*, mutations and/or SCNA's in *H3F3A*, *EGFR, CDK4/6*, *IDH, NF1,* and *FGFR3. NTRK* fusions are rare and have only been detected in 0.3% of cancers with a higher prevalence in GBM of 1.4% [63]. Given the degree of positive results with TRK inhibitors, *TRK* fusions are important to identify [5, 6]. At study onset, our institution participated in basket trials with rare gene fusions, as well as *BRAF* and *IDH* mutations. Specifically, we have an open phase 2 basket trial (NAVIGATE) with Larotrectinib (EudraCT: 2015‐003582‐28) that the patient with the *NTRK2* fusion has been included in. One patient was included in an early clinical trial based upon a *H3F3A* mutation. The results from these trials will be reported separately. The rare incidence of mutations and gene fusions results in a limited number of patients for the open trials for patients with GBM, but the genomic profiling program has attracted more trials. Trial availability is a dynamic process and recently a new trial opened at our institution with TMB as inclusion criteria (NCT03668119). International umbrella and basket trials for alterations found in our cohort do exist and are open for inclusion but the long travel distances to participate in a phase 1 trial can be difficult for these fragile patients. However, our study shows that it is possible to have genomic results ready at time of first progression, that GBM indeed does harbor alterations for targeted therapy, and that there is an unmet need for more local trials. Whenever possible, a relapse sample for a new genomic profile should be performed due to clonal evolution during treatment. Furthermore, the study underlines the necessity to set up international trials with adaptive designs to account for rare aberrations and for better cooperation, speed, and visibility. Majority of patients with GBM should enter clinical trials but a huge obstacle is the clinical deterioration that hinders participation in such. This translational focus with incorporation of molecular‐driven data for clinical trial designs has also been a priority in the neuro‐oncology community [64, 65]. Therefore, we should consider moving experimental treatment to 1st‐line therapy as is elegantly done in the N^2^M^2^ trial umbrella trial (NCT03158389) and was done in the CheckMate trials 209–498/548 (NCT02617589 and NCT02667587). Extensive analyses are possible and available [66, 67] but until whole‐genome sequencing/WES and RNA expression analyses can be performed at an acceptable turnaround time in the clinical setting, we suggest using panel sequencing to be able to use the genomic results for first‐line treatment. This also facilitates the possibility for a deeper sequencing than WES. What is equally important is not to treat patients with significantly known unfavorable markers and focus on quality of life. The project has changed clinical practice at our institution as we now offer a genomic profile for newly diagnosed patients and again at relapse, expanding the treatment options for these patients.

## 6 Conclusion

Our study shows feasibility of genomic profiling in GBM for therapeutic purposes. Noticeably, we identified one *NTRK2* fusion and found TMB‐high or CI‐median to be significantly correlated with worse survival. Based on the study results, we now offer a genomic profile for GBM patients at our institution at time of diagnosis and at relapse. The setup has changed from a research study to a clinical implementation, and trials are being planned. Based on the lack of patients' inclusion into targeted therapy trials, we propose a marker‐based approach in experimental adaptive trials already for the first‐line treatment. The molecular knowledge and technology are ahead of the clinical trials offered in GBM, and we foresee that future studies have a greater translational focus to make benefit of all the tremendous research already performed in this field.

### Strengths and limitations

Our study has the strength of being a prospective study with only newly diagnosed, treatment‐naïve GBM patients included, diagnosed after the 2016 WHO classification of brain tumors and with full clinical data. We had a multidisciplinary translational collaboration with all the specialties involved in GBM, surgeons, pathologists, radiologists, clinicians, Center for Genomic Medicine, and the Danish Cancer Society. Limitations were the small number of patients combined with the limited number of open trials for GBM patients at our institution and hence a minimal clinical utility of the results.

## Conflict of interest

The authors declare no conflict of interest.

## Author contributions

DSN, UL and HSP concepted the study. DSN included the patients, designed the clinical database, and wrote the manuscript. JS‐R and JB applied the specimens. PH headed the preparation of tissue in RNA‐later. FCN, CWY, OØ, and AYS headed the genomic analyses and wrote the reports. SK performed subgroup analyses. MHT performed the TMB analyses. UL, HSP, SRM, CWY, and OØ performed critical review of the manuscript, and all authors have read and approved of the manuscript.

## Supporting information


**Fig. S1.** Frequency plots of GBM cohort.
**Fig. S2.** Kaplan–Meier curves.
**Fig. S3.** Kaplan–Meier curves with MGMT‐status and OS in each subgroup, regardless of treatment.
**Fig. S4.** Kaplan–Meier curves with *GRB2* and *SMYD4* status and OS.
**Fig. S5.** Kaplan–Meier curve with numbers at risk for OS for the bad prognostic group (TMB‐high + CI‐median vs. the good prognostic group (TMB‐median/low + CI‐high + low).
**Fig. S6.** Histogram distribution.
**Table S1.** Filter list for 95 genes used for mutation call.
**Table S2.** A list of all tumor specific mutations called.
**Table S3.** Univariate analyses modelling the probability of selected genes and clinical variables as compared to OS.
**Table S4.** Fishers exact test for modelling the probability of completing the planned treatment.
**Table S5.** TMB and CI with the different variables (PS, age, steroid dose and MGMT‐status) shown.
**Table S6.** Gene set enrichments with chromosomal location among GBM subclasses and according to CI and TMB.

**Table S7.** Genelist of glioblastoma and chromosome localization.
