# Supplementary material for: Genomic profiling of newly diagnosed glioblastoma patients and its potential for clinical utility – a prospective, translational study
Source: Mol Oncol. 2020 Sep 18;14(11):2727–43. doi: 10.1002/1878-0261.12790 (PMC7607169; doi:10.1002/1878-0261.12790)
Supplement: Supplementary file 1 — Fig. S1. Frequency plots of GBM cohort. Fig. S2. Kaplan–Meier curves. Fig. S3. Kaplan–Meier curves with MGMT‐status and OS in each subgroup, regardless of treatment. Fig. S4. Kaplan–Meier curves with GRB2 and SMYD4 status and OS. Fig. S5. Kaplan–Meier curve with numbers at risk for OS for the bad prognostic group (TMB‐high + CI‐median vs. the good prognostic group (TMB‐median/low + CI‐high + low). Fig. S6. Histogram distribution. Table S1. Filter list for 95 genes used for mutation call. Table S2. A list of all tumor specific mutations called. Table S3. Univariate analyses modelling the probability of selected genes and clinical variables as compared to OS. Table S4. Fishers exact test for modelling the probability of completing the planned treatment. Table S5. TMB and CI with the different variables (PS, age, steroid dose and MGMT‐status) shown. Table S6. Gene set enrichments with chromosomal location among GBM subclasses and according to CI and TMB. [file MOL2-14-2727-s001.pdf]

ABCB1	CDX4	HEATR7B2	PIK3CA	SULT1B1
ABCC9	CHEK2	IDH1	PIK3RI	TCHH
ADAM29	COL1A2	IDH2	PLCH2	TERT
AFM	CFAP47	IL18RAP	PODNL1	TMEM147
ALK	DCAF12L2	KEL	POT1	TP53
ANKRD36	DRD5	KMT2C	PTEN	TPTE2
ATRX	DYNC1I1	KRTAP20-2	QKI	TRPV6
BCOR	EGFR	LCE4A	RB1	UGT2a3
BRAF	ERBB1	LRRC55	RFX6	VEGFA
BRCA1	ERBB2	LUM	RPL5	WNT2
BRCA2	ERBB3	LZTR1	SCN9A	ZNF844
GCSAML	ERBB4	MET	SEMA3C	ZNF99
CALCR	FGA	MMP13	SEMA3E	ATM
CARD6	FOXR2	NF1	SEMG1	MLH1
CD3EAP	FRMD7	NLRP5	SIGLEC8	MSH2
CDH18	GABRA1	NOTCH	SLC26A3	MSH6
CDH9	GABRA6	ODF4	SPRYD5	PMS2
CDHR3	GPX5	PARD6B	SPTA1	POLE
CDKN2A	H3F3A	PDGFRA	STAG2	H3F3B

Table S1. Filter list for 95 genes used for mutation call.

**Gene**	**Variant**	**Variant**	**Variant**
ABCB1	c.3262G>A; p.D1088N	c.1738C>T; p.R580W	
ABCC9	c.2599G>A; p.V867I		
ADAM29	**c.1043G>A; p.R348H**	c.731T>C; p.L244S	
ATRX	c.6332G>A; p.R2111Q	**c.3967G>T; p.E1323***	**c.6895_6896delCC; p.P2299fs*22**
	c.5408G>A; p.R1803H	**c.4269_4272delGAAA; p.K1424fs*65**	**c.1507C>T; p.Q503***
	**c.6889_6890delTT; p.L2297fs*24**	**c.4809G>T; p.Q1603H**	
AFM	c.433G>A; p.E145K	c.1954delG; p.D652fs*8	c.4049G>T; p.G1350V
BCOR	c.3874G>T; p.E1292*	c.3800_3801dupCA; p.G1268fs*68	
BRCA2	c.3847_3848delGT; p.V1283fs*2		
BRAF	**c.1786G>C; p.G596R**		
CFAP47	**c1660C>T; p.R554C**		
CALCR	c.265G>T; p.V89L		
CDH9	c.2307C>A; p.D769E	c.2307C>A; p.D769E	
CDKN2A	**c.250G>A; p.D84N**	**c.193G>A; p.G65S**	
CDH18	c.1890_1892delGGT; p.V631del		
CDX4	c.625A>C; p.N209H		
CFAP47	c.7400T>A; p.L2467Q	c.3827A>G; p.Y1276C	
COL1A2	c.1558G>T; p.G520C	c.3103G>A; p.A1035T	
EGFR	c.865G>A; p.A289T	**c.787A>G; p.T263P**	c.664C>T; p.R222C
	**c.866C>T; p.A289V (x3)**	**c.2156G>C; p.G719A**	**c.1793G>T; p.G598V**
	**c.1793G>T; p.G598V**	c.1934C>G; p.S645C	**c.754C>T; p.R252C**
	c.2006G>A; p.R669Q	**c.664C>T; p.R222C**	
ERBB4	**c.2777C>T; p.T926M**		
GABRA6	c.198T>A; p.S66R	c.1223C>T; p.S408L	
GABRB2	c.442G>T; p.V148F		
H3F3A	**c.83A>T; p.K28M**		
IDH1	**c.395G>A; p. R132H (x5)**		
IL18RAP	c.475G>A; p. A159T	c.1234G>A; p.V412I	
KEL	c.1283G>A; p.R428H		
KMT2C	c.1555C>G; p.H519D	c.2015A>G; p.E672G	
LZTR1	c.727T>C; p.F243L	c.467A>G; p.K156R	
MET	c.2533C>A; p.L845I	c.1579A>C; p.S527R	
MMP13	c.120+1G>T	c.998G>A; p.R333H	
MSH2	**c.1735A>T; p.K579***		
NF1	**c.3479delG; p.G1160fs*6**	**c.479+2T>G (splice site)**	**c.5565_5567delTCT; p.L1856del**
	**C.3739_3742delTTTG; p.F1247fs*18**	**c.4108C>T; p.Q1370***	**c.1318C>T; p.R440***
	c.4169T>G; p.L1390R	**c.3861_3862delCT; p.F1287fs*26**	c.4157delA; p.K1386fs*20
	**c.5902C>T; p.R1968***	**c.7996_7997delAG; p.S2666fs*5**	**c.1888delG; p.V630***

	**c.7348C>T; p.R2450***	**c.3916C>T; p.R1306***	**c.1746CA>; p.C582***
	c.1527+1_1527+4delGTAA; p.Y2264fs*5	**c.6789_6792delTTAC; p.Y2285fs*5**	**c.3089C>A; p.S1030***
	**c.7996_7997delAG; p.S2666fs*5**		
NLRP5	c.509G>A; p.G170E	c.1552C>T; p.R518C	
NOTCH1	c.2380G>C; p.E794Q	c.3317A>T; p.Q1106L	
PDGFRA	c.862T>A; p.Y288N	c.1607T>A; p.V536E	**c.868T>C; p.C290R**
PIK3CA	c.1638G>T; p.Q546H	**c.1030G>A; p.V344M**	**c.263G>A; p.R88Q**
	**c.1634A>G; p.E545G**	c.1134T>G; p.C378W	**c.353G>A; p.G118D**
	**c.3139C>T; p.H1074Y**	**c.277C>T; p.R93W**	
PIK3R1	**c.584delG; p.R195fs*15**	c.918-920delGAG; p.R307del	**c. 880A>G; p.N294D**
	**c.827_829delCGA; p.T276del**	c.1691A>G; p.N564S	**c.483dupA; p.R162fs*5**
	**c.1690A>G; p.N564D**	c.918_920delGAG; p.R307del	c.869A>G; p.D290G
	**c.316G>A; p.g106R**	c.543_545delATA; p.E181_Y182delinsD	c.937T>C; p.W313R
	c.918_920delGAG; p.R307del		
PLCH2	c.3571C>T; p.R1191C	c.1622T>C; p.V541A	
PTEN	**c.388C>G; p.R130G**	**c.301dupA; p.I101fs*6**	**c.1133_1136delGATA: p.R378fs**
	**c.517C>T; p.R173C**	**c.302T>C; p.I101T**	**c.377C>G; p.A126G**
	**c.492+1G>T (splice site)**	**c.170delT; p.L57fs*42**	**c.1113delC; p.D371fs**
	**c.209+1G>A (splice site)**	**c.385G>T; p.G129***	**c.72C>G; p.D24E**
	**c.517C>T; p.R173C**	**c.989_990delAA; p.K330fs*12**	c.737C>T; p.P246L
	**c.479C>A; p.T160N**	**c.139A>G; p.R47G**	**C800delA; p.K267fs*9**
	**c.388C>G; p.R130G (x2)**	**c.820delT; p.W274fs*2**	**c.728+1_728+4delGTAA (splice site)**
	**c.567dupA; p.P190fs*12**	c.209+1_209+4delGTAA	**c.333G>A; p.W111***
	**c.955_958delACTT; p.T319***	c.166T>G; p.F56V	c.518G>A; p.R173H
	c.373A>G; p.K125E	**c.955_958delACTT; p.T319***	**c.87T>G; p.Y29***
	**c.610C>A; p.P204T**	**c.209+1209+4delGTAA**	**c.987_990delTAAA; p.N329fs*14**
	**c.945T>A; p.Y315***	c.466G>A; p.G156R	c.98T>C; p.I33T
	**c.907delA; p.I303fs*4**	**c.1007dupA; p.Y336***	**c.212G>A; p.C71Y**
	**c.389G>A; p.R130Q**	**c.697C>T; p.R233***	
RB1	**c.368dupA; p.N123fs*8**	**c.446C>G; p.S149***	**c.1422-1G>C (splice site)**
	**c.1494T>G; p.Y498***	**c.763C>T; p.R255***	**c.718A>T; p.K240***
	**c.2520+1G>A**	**c.1422-1G>C (splice site)**	**c.264+1G>C (splice site)**
	**c.2663G>A; p.S888N**	**c.1575delC; p.F526fs*6**	**c.277C>T; p.Q93***
	**c.1499-1G>A (splice site)**		
RPL5	**c.125delA; p.N42fs*10**	**c.3+1G>A (splice site)**	
SCN9A	c.1901G>A; p.R634H	c.326T>C; p.L109P	c.3541A>G; p.S1181G
	c.1502C>T; p.S501L		
SEMA3C	c.328-1G>A		
SLC26A3	c.1697G>A; p.R566Q		
STAG2	**c.2285_2289delAGAAA; p.K762fs*21**	**c.913C>T; p.R305***	
SULT1B1	c.824A>T; p.E275V		
TCHH	c.4322G>A; p.R1441H	c.2533C>T; p.R845C	c.513G>C; p.E1713Q
	c.2318C>T; p.A773V	**c.682C>T; p.Q228***	**c.4873G>A; p.E1625K**
TERT	c.3116C>T; p.T1039M		
TP53	c.422G>A; p.C141Y	**c.292C>T; p.P98S**	**c.1024C>T; p.R342***
	**c.733G>A; p.G245S**	**c.646G>A; p.V216M**	**c.817C>T; p.R273C**
	**c.451C>T; p.P151S**	**c.916C>T; p.R306***	**c.749C>T; p.P250L**
	**c.713G>A; p.C238Y**	**c.427G>A; p.V143M**	**c.649delG; p.V217fs*30**
	**c.445dupT; p.S149fs*32**	**c.818G>A; p.R273H**	**c.725G>T; p.C242F**
	**c.844C>T; p.R282W**	**c.473G>A; p.R158H**	**c.814G>A; p.V272M**
	**c.963dupA; p.322fs*15**	**c.517G>C; p.V173L**	**c.772G>T; p.E258***
	**c.746G>T; p.R249M**	**c.853G>A; p.E285K**	**c.1023_1024delCC; p.F341fs*5**
	**c.434T>G; p.L145R**	**c.817C>T; p.R273C**	**c.587G>C; p.R169P**
	**c.200delC; p.P67fs*56**	**c.527G>A; p.C176Y**	**c.1024C>T; p.R342***
	**c.592G>T; p.E198***	**c.712T>A; p.C238S**	**c.770T>A; p.L257Q**
	**c.934_935dupAC; p.S313fs*33**	**c.653_654delTG; p.V218fs*3**	**c.524G>A; p.R175H**
	**c.818G>A; p.R273H**	**c.832C>A; p.P278T**	**c.712T>A; p.C238S**
	**c.653_654delTG; p.V218fs*3**	**c.814G>A; p.R175H**	
TRPV6	c.536G>A; p.R179H		
UGT2A3	c.350T>C; p.I117T		
WNT2	c.89T>A; p.M30K		

Table S2. A list of all tumor specific mutations called. Mutations in **bold** were defined as pathological. For definition, see Material and Method section.

**Covariate**	**HR (95 % CI)**	***P*-value**
Genes (2-fold change)		
TERT promotor region	1.62 (0.68-3.85)	0.275
PTEN	0.71 (0.30-1.67)	0.438
CDKN2A/B	1.00 (0.50-1.20)	0.996
EGFR	0.94 (0.55-1.62)	0.831
MGMT-WT	0.31 (0.17-0.58)	0.0002*
RB1	0.64 (0.36-1.14)	0.127
NPAS3	0.64 (0.34-1.22)	0.175
AKT1	0.69 (0.36-1.30)	0.248
IRS2	0.67 (0.36-1.28)	0.228
QKI	1.06 (0.58-1.94)	0.860
TP53	0.58 (0.28-1.19)	0.136
CDK4	0.66 (0.33-1.31)	0.232
CDKN2C	0.73 (0.33-1.60)	0.426
SMYDA	0.32 (0.12-0.89)	0.029*
MDM2	0.56 (0.25-1.23)	0.147
NF1	0.81 (0.35-1.89)	0.621
PDGFRA	0.58 (0.21-1.62)	0.300
PRDM2	0.55 (0.17-1.77)	0.314
MYC	0.53 (0.23-1.25)	0.148
MDM4	0.56 (0.20-1.55)	0.265
GRB2	0.23 (0.06-0.94)	0.040*
CCND2	0.37 (0.12-1.19)	0.097
HYDIN	0.84 (0.26-2.32)	0.734
LSAMP	0.72 (0.26-2.02)	0.535
CDKN2A	1.89 (0.44-8.16)	0.392
FGFR3	0.23 (0.03-1.70)	0.150
AKT3	0.26 (0.04-1.90)	0.184
ATRX	1.00 (0.31-3.21)	0.995
CCNE1	1.07 (0.26-4.41)	0.928
IDH1	0.24 (0.03-1.76)	0.161
**Clinical variables**		
Age at diagnosis (≥70 years *vs.* < 70 years)	0.39 (0.22-0.70)	0.001*
Corticosteroid use (yes *vs.* no)	0.56 (0.32-0.98)	0.04*
WHO performance status (≥ 2 *vs.* 0-1)	0.40 (0.20-0.81)	0.01*
Completes aggressive treatment (yes *vs.* no)	2.32 (1.33-4.04)	0.003*

Table S3: Univariate analyses modelling the probability of selected genes and clinical variables as compared to overall survival (OS). Fishers exact test.

**Covariate**	***P*-value**
Genes (2-fold change)	
TERT promotor region	0.88
MGMT-WT	0.02*
CDKN2A/B	0.36
EGFR	0.47
CDK4	0.71
PDGFRA	1.00
MDM4	0.68
PTEN	1.00
MDM2	0.30
IDH1	0.15
CDKN2A	1.00
MET	1.00
MYC	1.00
RB1	0.40
CCND2	0.40
CDKN2C	1.00
AKT1	1.00
AKT3	0.40
FGFR2	0.40
NPAS3	0.40
PRDM2	1.00
QKI	1.00
Clinical variables	
Age at diagnosis (≥70 years *vs.* < 70 years)	0.51
Corticosteroid use (yes *vs.* no)	0.09
WHO performance status (≥ 2 *vs.* 0-1)	0.08

Table S4. Fishers exact test for modelling the probability of completing the planned treatment. *N* = 77 whereof 31 has completed radiotherapy/Temozolomide and adjuvant Temozolomide and 46 has not. Only genes with biallelic losses or amplifications are shown.

A

Variables	p-value	Hazard ratio	95% confidence interval
**TMB high *vs* low/median**	0.005	2.87	1.38-5.97
**CI median *vs* high/low**	0.04	1.78	1.04-3.04

B

Variables	p-value	Hazard ratio	95% confidence interval
**TMB high *vs* low/median**	0.009	3.29	1.35-8.02
**PS 0-1 *vs* ≥ 2**	0.48	0.71	0.27-1.84
**Age < 70 *vs* ≥ 70**	0.01	0.41	0.21-0.81
**Steroid (mg) ≤ 10 *vs* > 10**	0.22	0.67	0.35-1.27
**MGMT-methylated *vs* wild-type**	0.00005	3.82	2.0-7.29

Variables	p-value	Hazard ratio	95% confidence interval
**CI median *vs* low/high**	0.13	1.51	0.88-2.60
**PS 0-1 *vs* ≥ 2**	0.01	0.37	0.18-0.80
**Age < 70 *vs* ≥ 70**	0.001	0.33	0.17-0.62
**Steroid (mg) ≤ 10 *vs* > 10**	0.53	0.82	0.44-1.52
**MGMT-methylated *vs* wild-type**	0.00003	4.04	2.10-7.77

Table S5. Tumor mutational burden (TMB) and chromosomal instability (CI) with the different variables (performance status (PS), age, steroid dose and MGMT-status) shown. Assessed using univariate (A) and multivariate (B) Cox proportional hazard models.

TMB High versus Median and Low				
Gene Symbol	p-value	q-value	Fold change	Chromosomal Loc.
EBF1	0,00	1,00	2,2	chr5q34
LOC105377656	0,02	1,00	2,2	---
GAS2	0,02	1,00	2,2	chr11p14.3
FSTL5	0,04	1,00	2,1	chr4q32.3
GPX3	0,04	1,00	1,9	chr5q33.1
DSP	0,01	1,00	1,8	chr6p24
DLX5	0,04	1,00	1,8	chr7q22
DACH1	0,05	1,00	1,7	chr13q22
LHX8	0,01	1,00	1,7	chr1p31.1
IGF1	0,02	1,00	1,7	chr12q23.2
CACNA2D1	0,03	1,00	1,6	chr7q21-q22
APELA	0,01	1,00	1,6	chr4q32
DLX1	0,02	1,00	1,6	chr2q32
KANK4	0,00	1,00	1,6	chr1p31.3
DLX2	0,00	1,00	1,6	chr2q32
MATN3	0,00	1,00	1,6	chr2p24-p23
DPT	0,04	1,00	1,6	chr1q12-q23
TMEM119	0,05	1,00	1,5	chr12q23.3

GSEA - TMB High versus Median and Low						
Geneset	Size	Matches	ES	NES	p	q
HALLMARK_TGF_BETA_SIGNALING	54	54	0,6	1,9	0,00	0,1
HALLMARK_HEDGEHOG_SIGNALING	36	36	0,5	1,7	0,01	0,3
HALLMARK_WNT_BETA_CATENIN_SIGNALING	42	41	0,4	1,4	0,08	1

CI Median versus Low and High				
Gene Symbol	p-value	q-value	Fold change	Chromosome Loc.
SLITRK3	0,00	0,66	2,0	chr3q26.1
TRDC	0,05	0,75	2,0	chr14q11
VEGFA	0,00	0,66	1,9	chr6p12
IGFBP5	0,00	0,58	1,9	chr2q35
CPNE4	0,02	0,72	1,8	chr3q22.1
HLA-DRB1	0,04	0,75	1,8	chr6p21.3
ART3	0,01	0,72	1,7	chr4q21.1
FAM19A1	0,05	0,75	1,7	chr3p14.1
PLPPR5	0,02	0,72	1,6	chr1p21.3
ADM	0,04	0,75	1,6	chr11p15.4
GRB14	0,02	0,72	1,6	chr2q22-q24
TMEM178A	0,01	0,72	1,6	chr2p22.1
TNFAIP6	0,03	0,74	1,6	chr2q23.3
FOXG1	0,01	0,72	1,5	chr14q13

GSEA - CI Median versus Low and High						
Geneset	Size	Matches	ES	NES	p	q
HALLMARK_CHOLESTEROL_HOMEOSTASIS	74	72	0,51	1,77	0,02	0,3
HALLMARK_APICAL_JUNCTION	200	192	0,38	1,47	0,07	1
HALLMARK_WNT_BETA_CATENIN_SIGNALING	42	41	0,41	1,38	0,09	1

Table S6. Gene set enrichments with chromosomal location among glioblastoma subclasses and according to chromosomal instability (CI) and tumor mutational burden (TMB). Enrichments were examined in the molecular signatures database (MSig) among the all curated gene sets (tumor classes) or the HALLMARK gene sets (CI and TMB). The uncorrected NES, p and q values are indicated.

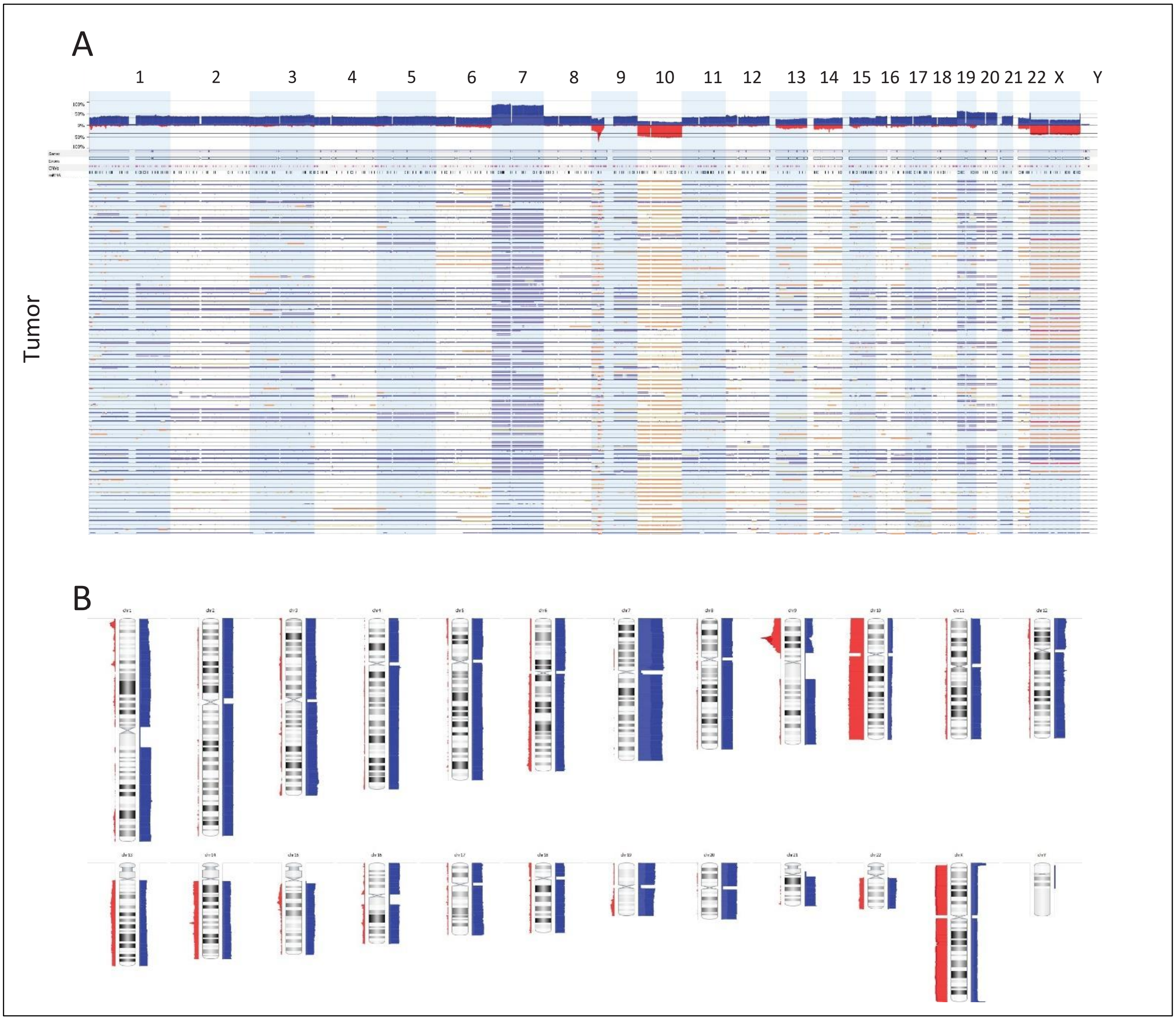

Figure S1. Frequency plots of glioblastoma cohort.

A: Frequency plot of the cohort (upper plot) and frequency plots for individual samples. Blue marking indicates gains/amplification and red marking indicates deletions. Yellow marking of plots for individual samples marks LOH areas. The most common chromosomal aberrations in the glioblastoma cohort was gain of chromosome 7 (>80%), deletion of 9p with biallelic deletion in area containing *CDKN2A/B* (>80%) and deletion of chromosome 10 including *PTEN* on the q-arm of the chromosome (>50%).

B: Alternative frequency plot displaying chromosomal aberrations for the glioblastoma cohort on individual chromosomes. Marking and conclusion as for A.

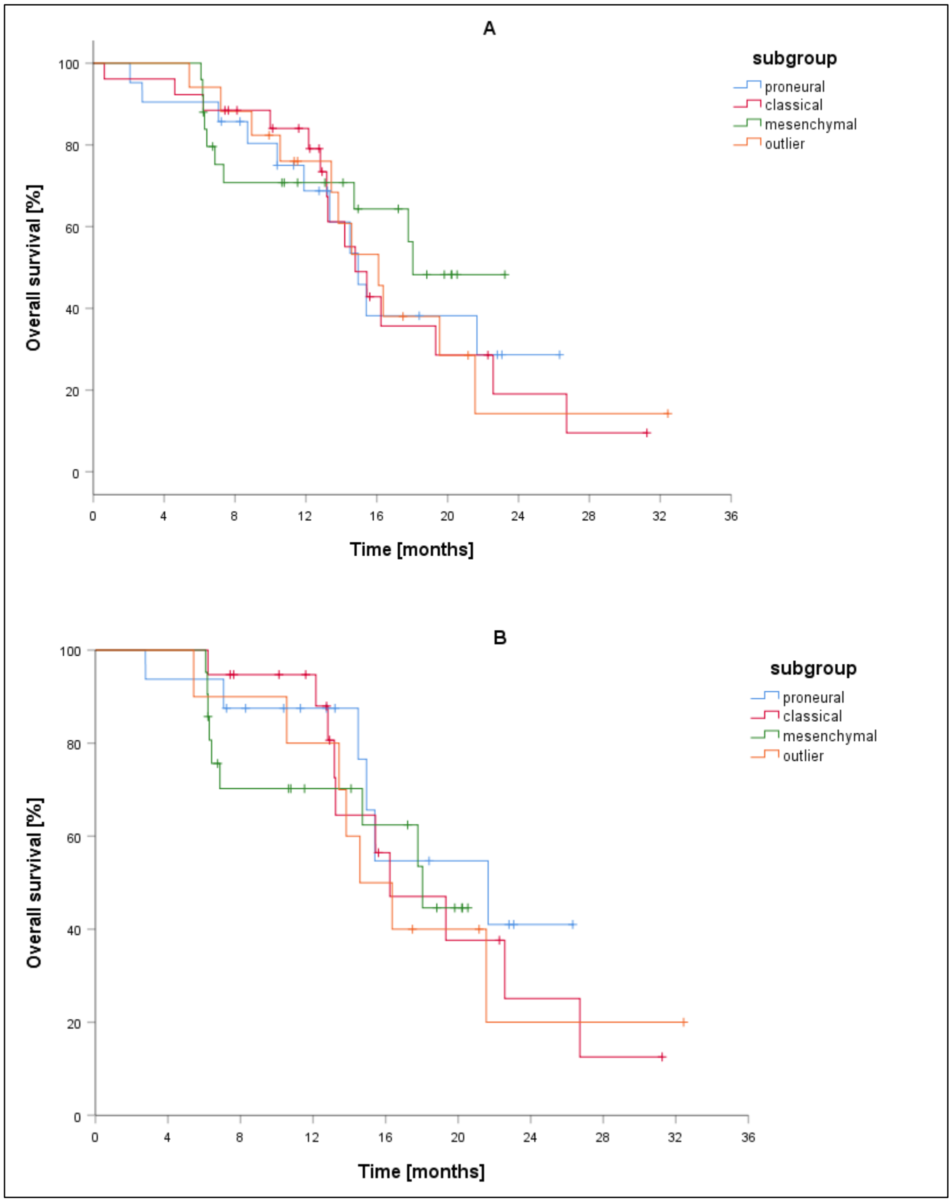

Figure S2A-B. Kaplan Meier curves. Overall survival and subgroup distribution.
A: Regardless of treatment N = 89. No difference in overall survival and subgroup distribution. P = 0.85
B: In the TMZ/RT treated group. N = 66. No difference in overall survival and subgroup distribution. P = 0.84

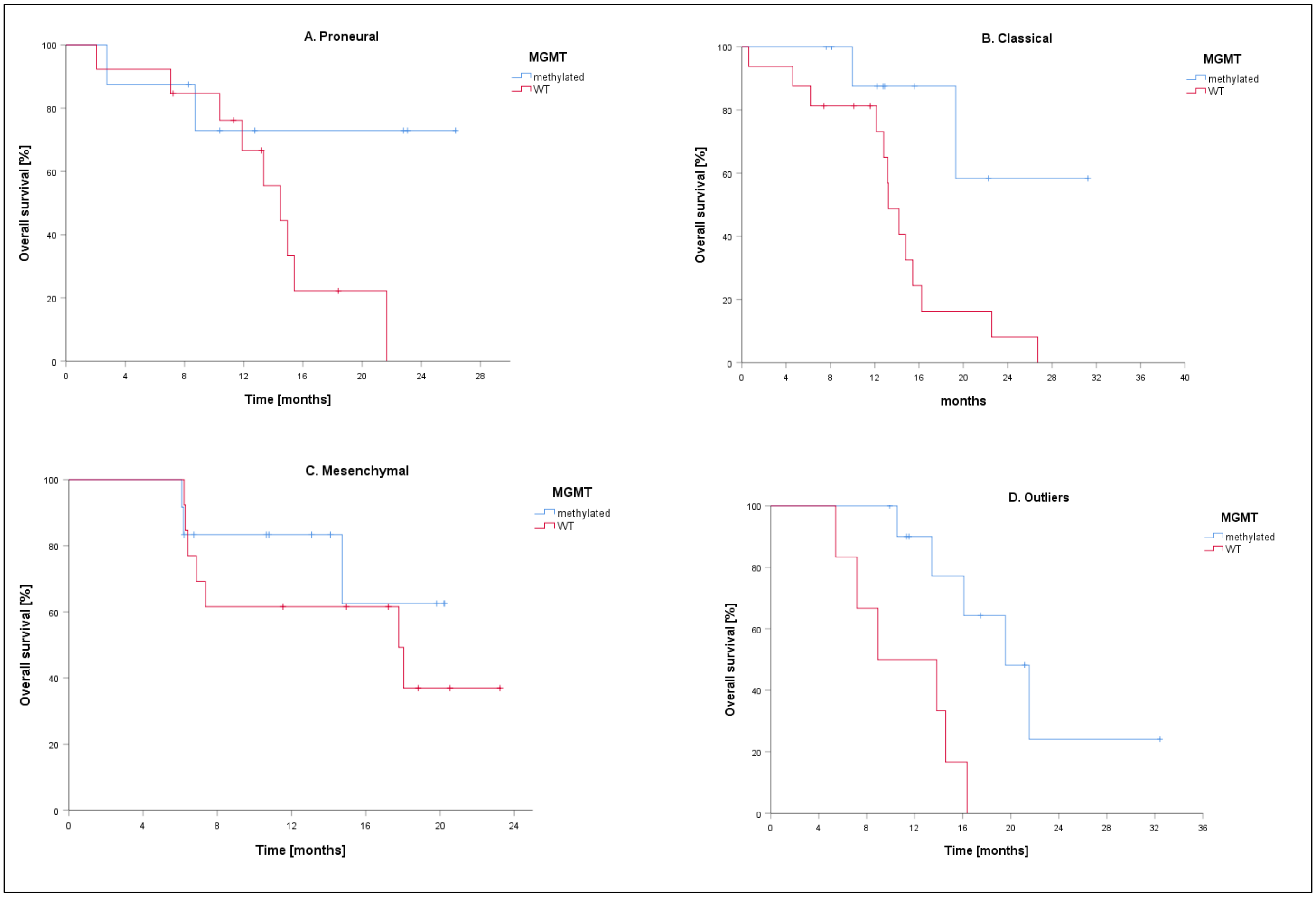

Figure S3A-E. Kaplan-Meier curves with MGMT-status and overall survival (OS) in each subgroup, regardless of treatment.
A: Proneural. N = 21 (MGMT-WT, N=13/ MGMT-meth, N =8). Median OS of 14.5 months *vs.* not reached. P = 0.11. (95% CI: 12.7-17.2).
B: Classical. N = 26 (MGMT-WT, N=16 / MGMT-meth, N=10). Median OS of 13.2 months *vs.* not reached. P = 0.02. (95% CI: 12.4-17.2).
C: Mesenchymal. N = 25 (MGMT-WT, N=13 / MGMT-meth, N=12). Median OS of 17.8 *vs.* not reached. P = 0.39. (95% CI: 4.7-30.9).
D: Outlier. N = 17 (MGMT-WT, N=6 / MGMT-meth, N=11). Median OS of 19.5 (95% CI: 14.6-24.4) *vs.* 8.9 months, respectively. P = 0.005 (95% CI: 1.0-16.9).

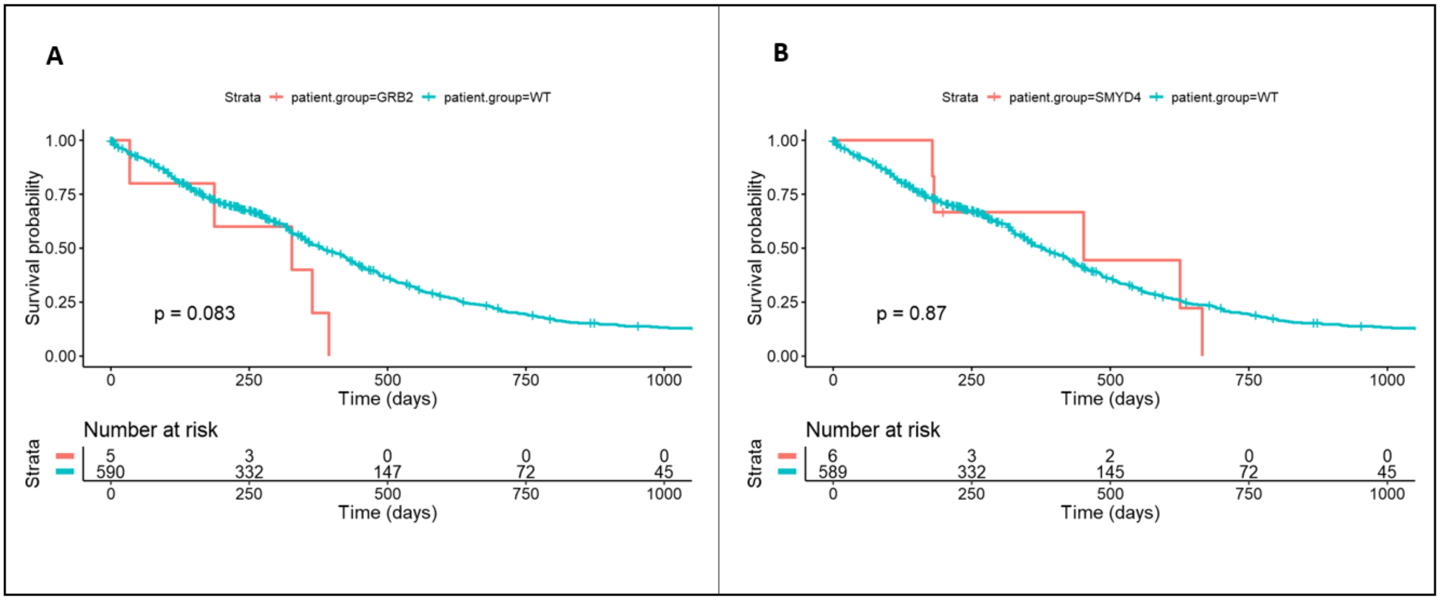

Figure S4A-B. Kaplan-Meier curves with *GRB2* and *SMYD4* status and overall survival (OS). Data from the TCGA dataset.
A: *GRB2* mutation was identified in 5/596 samples. A trend towards a worse OS was found in the mutated samples. P = 0.08.
B: *SMYD4* mutation was identified in 6/596 samples. No difference in OS was found. P = 0.87.

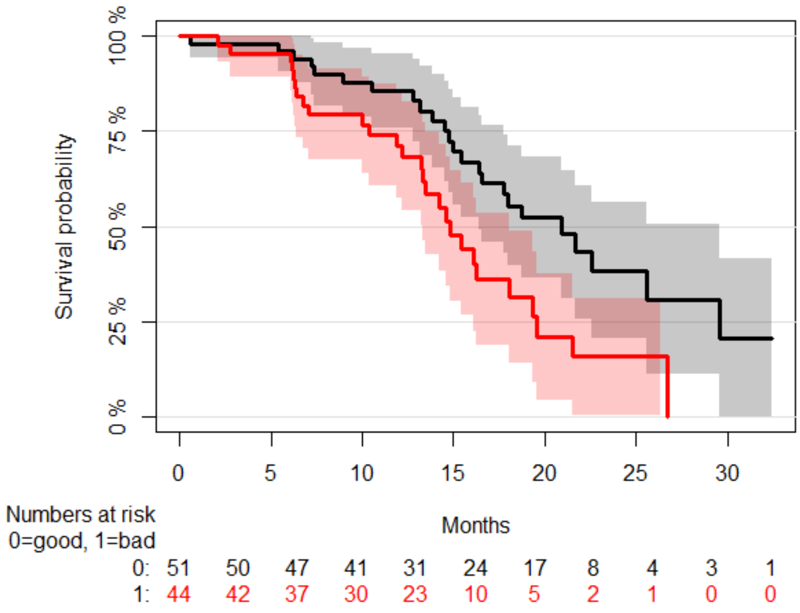

Figure S5. Kaplan-Meier curve with numbers at risk for overall survival (OS) for the bad prognostic group (tumor mutational burden (TMB)-high + chromosomal instability (CI)-median *vs.* the good prognostic group (TMB-median/low + CI-high + low).
The bad prognostic group had a significantly worse OS of 14.8 months (95% CI: 12.4-17.1) *vs.* 20.9 months (95% CI: 15.9-25.9), respectively (p=0.008).

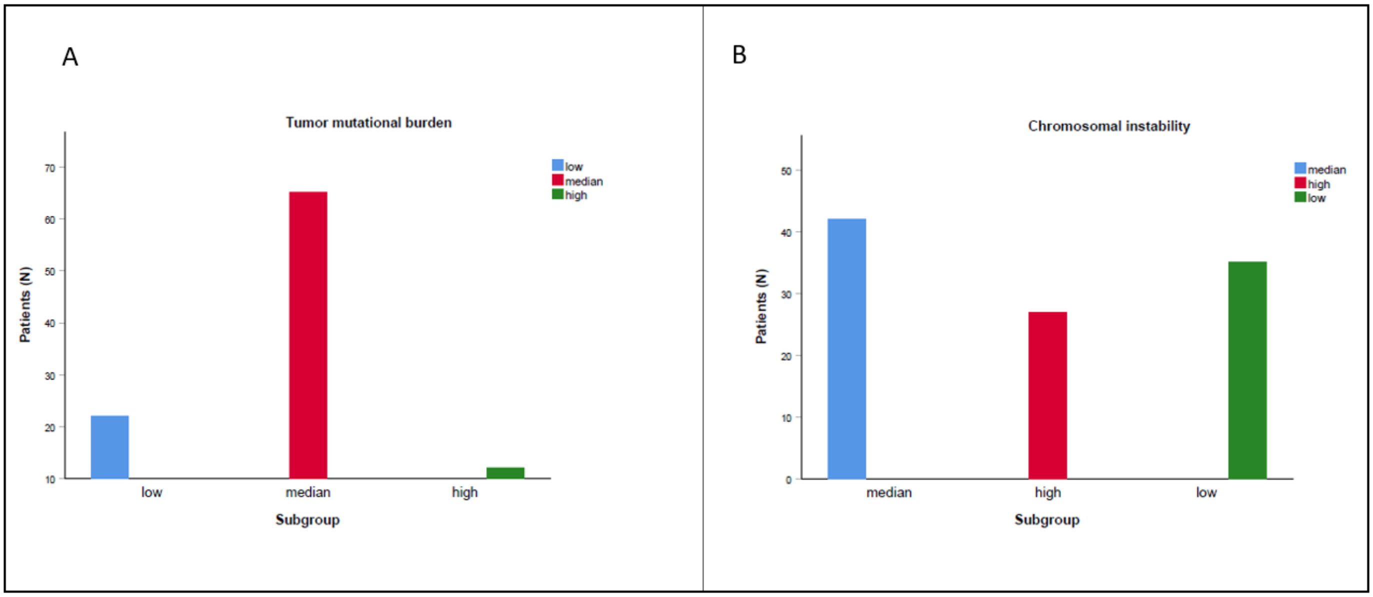

Figure S6. Histogram distribution.
A: Tumor mutational burden (TMB) with TMB-low (0-1.5, *N*=22), TMB-median (1.6-2.9, *N*=65) and TMB-high (≥3.0, *N*=12). Total *N*=99.
B: Chromosomal instability (CI) with CI-low (0-7 segmental chromosomal aberrations (SCA), *N*=35), CI-median (8-15 SCA, *N*=42) and CI-high (>15 SCA or aneuploid background, *N*=27). Total *N*=104
